# Establishment of age- and sex-adjusted reference data for hand bone mass and investigation of hand bone loss in patients with rheumatoid arthritis treated in clinical practice: an observational study from the DANBIO registry and the Copenhagen Osteoarthritis Study

**DOI:** 10.1186/s13075-016-0952-y

**Published:** 2016-02-24

**Authors:** Lykke Midtbøll Ørnbjerg, Mikkel Østergaard, Trine Jensen, Lars Hyldstrup, Pernille Bach-Mortensen, Pernille Bøyesen, Anja Thormann, Ulrik Tarp, Wolfgang Peter Bøhme, Hanne Lindegaard, Uta Engling Poulsen, Anette Schlemmer, Niels Graudal, Anne Rødgaard, Jakob Espesen, Gina Birgitte Kollerup, Bente Glintborg, Ole Rintek Madsen, Dorte Vendelbo Jensen, Merete Lund Hetland

**Affiliations:** DANBIO registry and Copenhagen Center for Arthritis Research, Center for Rheumatology and Spine Diseases, Centre of Head and Orthopedics, Ndr. Ringvej 57, DK-2600 Glostrup, Denmark; Rigshospitalet, Department of Clinical Medicine, Faculty of Health and Medical Sciences, University of Copenhagen, Copenhagen, Denmark; Department of Endocrinology, Hvidovre Hospital, Hvidovre, Denmark; Department of Rheumatology, Diakonhjemmet Hospital, Oslo, Norway; Department of Rheumatology, Aarhus University Hospital, Aarhus, Denmark; Department of Medicine, Hospital of South West Jutland, Esbjerg, Denmark; Department of Rheumatology, Odense University Hospital, Odense, Denmark; Rheumatism Hospital, University of Southern Denmark, Gråsten, Denmark; Department of Rheumatology, Aalborg University Hospital, Aalborg, Denmark; Department of Infectious Diseases and Rheumatology, Copenhagen University Hospital Rigshospitalet Blegdamsvej, Copenhagen, Denmark; Center for Rheumatology and Spine Diseases, Rigshospitalet Glostrup, Glostrup, Denmark; Department of Internal Medicine, Lillebælt Hospital, Vejle, Denmark; Department of Rheumatology, Copenhagen University Hospital Bispebjerg and Frederiksberg, Copenhagen, Denmark; Department of Internal Medicine, Holbæk Hospital, Holbæk, Denmark; Department of Medicine and Rheumatology, Copenhagen University Hospital Gentofte, Gentofte, Denmark; Department of Rheumatology, Nordsjællands Hospital, Hillerød, Denmark

**Keywords:** Rheumatoid arthritis, Bone mineral density, Anti-TNF

## Abstract

**Background:**

Rheumatoid arthritis is characterised by progressive joint destruction and loss of periarticular bone mass. Hand bone loss (HBL) has therefore been proposed as an outcome measure for treatment efficacy. A definition of increased HBL adjusted for age- and sex-related bone loss is lacking. In this study, we aimed to: 1) establish reference values for normal hand bone mass (bone mineral density measured by digital x-ray radiogrammetry (DXR-BMD)); and 2) examine whether HBL is normalised in rheumatoid arthritis patients during treatment with tumour necrosis factor alpha inhibitors (TNFI).

**Methods:**

DXR-BMD was measured from hand x-rays in a reference cohort (1485 men/2541 women) without arthritis randomly selected from an urban Danish population. Sex- and age-related HBL/year was estimated. DXR-BMD was measured in rheumatoid arthritis patients (n = 350: at start of TNFI, and ~2 years after TNFI start), of which 135 patients had three x-rays (~2 years prior to TNFI, at start of TNFI, and ~2 years after TNFI start). Individual HBL/year prior to and during TNFI was calculated and compared to reference values.

**Results:**

Estimated HBL/year varied strongly with age and sex. Compared to the reference values, 75 % of 135 patients had increased HBL prior to TNFI treatment and 59 % had increased HBL during TNFI treatment (*p* = 0.17, Chi-squared). In 38 % (38/101) of patients with increased HBL, HBL was normalised during TNFI treatment, whereas 47 % (16/34) of patients with normal HBL prior to TNFI had increased HBL during TNFI treatment. In the 350 patients, increased HBL during TNFI was associated with time-averaged 28-joint disease activity score (odds ratio 1.69 (95 % Confidence Interval 1.34-2.15)/unit increase, *p* < 0.001), and patients in time-averaged remission had lower HBL than patients without remission (0.0032 vs. 0.0058 g/cm^2^/year; *p* < 0.001, Mann-Whitney).

**Conclusions:**

We established age- and sex-specific reference values for DXR-BMD in a large cohort without arthritis. HBL was increased in the majority of rheumatoid arthritis patients initiating TNFI in clinical practice, and only normalised in a minority during TNFI.

**Electronic supplementary material:**

The online version of this article (doi:10.1186/s13075-016-0952-y) contains supplementary material, which is available to authorized users.

## Background

Bone involvement in rheumatoid arthritis (RA) has three hallmarks: localised destruction of joint bone, periarticular bone loss and generalised bone loss. The two former features are visible on x-rays as erosions and periarticular osteoporosis and are suggested to be caused by the same pathophysiologic pathway [1, 2]. Increased hand bone loss (HBL) measured by digital x-ray radiogrammetry (DXR) may precede the development of erosions, takes place throughout the disease course, and occurs in patients with no progression of radiographic joint damage [3–5]. Thus, HBL has been proposed as a more sensitive outcome measure for monitoring disease progression and treatment effect than the traditional evaluation of radiographs [5].

A common definition of increased HBL has not yet been established, which limits the clinical value of HBL and comparison between studies. Attempts to define increased HBL include HBL above the median in the study population, and the smallest detectable difference of HBL or cut-off values provided by the manufacturer based on the distribution of HBL in two early RA cohorts [6–10]. However, the normal HBL in healthy persons, shown to vary considerably with sex and age, has so far not been included in the definition of increased HBL [11, 12].

Impact of tumour necrosis factor alpha inhibitors (TNFI) on HBL in RA patients has been investigated in the PREMIER and BEST randomised controlled trials (RCTs) in early RA, where patients treated with TNFI in combination with methotrexate (MTX) had a significantly lower HBL compared with MTX monotherapy [5, 13]. No studies have analysed whether HBL is normalised during TNFI treatment.

The primary aims of the present study were: 1) to establish a large reference material for hand bone mass measured by DXR in order to estimate sex- and age-related HBL/year; and 2) to determine whether HBL in RA patients treated in clinical practice normalises during treatment with TNFI when switched from conventional synthetic disease-modifying anti-rheumatic drugs (csDMARDs) due to an unsatisfactory clinical response.

Secondarily, in RA patients treated in clinical practice, we aimed to: 3) investigate the association between HBL and radiographic progression during treatment with csDMARDs and TNFI; 4) investigate the association between HBL and inflammatory activity during TNFI; and 5) identify predictors of increased HBL during TNFI.

## Methods

### Reference cohort

The Copenhagen City Heart Study is a health survey of an adult urban Danish population selected by a random social security number algorithm. From 1991 to 1994, 10,135 participants were included and from this main cohort 1533 men and 2618 women without inflammatory joint disease had x-rays of the pelvis, knees, lumbar spine, hands and wrists performed once (Copenhagen Osteoarthritis Study) [14]. Age, sex and hand x-ray films were used in the present study.

### Patients with RA

In the Danish DANBIO registry, disease outcomes are reported prospectively in RA patients treated in clinical practice. DANBIO covers >90 % of TNFI treated RA patients [15].

We included all RA patients in DANBIO who: 1) were TNFI naïve; 2) started treatment with adalimumab, etanercept or infliximab before 1 July 2007; and 3) had at least two relevant hand x-rays (baseline and follow-up). A baseline x-ray had to precede the initiation of TNFI by less than 3 months, while the follow-up x-ray had to be obtained >6 months after the baseline x-ray. If available, a pre-baseline x-ray, which preceded both TNFI start and baseline x-ray with >6 months was collected. Five hundred and seventeen patients had three available x-rays, while 930 patients had two x-rays (baseline and follow-up) [16, 17].

### Ethical approval and patient consent

The individuals in the reference cohort signed an informed consent form. The Danish ethics committee for the City of Copenhagen and Frederiksberg approved the Copenhagen City Heart Study (100.2039/91).

The data from the patient cohort originates from the nationwide Danish DANBIO registry. DANBIO has been approved by The Danish Data Registry since the year 2000 (j. nr. 2007-58-0014 and j.nr. 2007-58-0006), and since October 2006 as a national quality registry by the National Board of Health (j. nr. 7-201-03-12/1). According to Danish law, informed consent and ethical approval were not required for the present study.

### Radiographic assessment of patient radiographs

Patient x-rays were available and collected as x-ray films or digital images. To facilitate reading, x-ray films were scanned and converted to a digital format. Reading was performed by an experienced reader, blinded to patient identity and image sequence, according to the modified Sharp method [18].

Annual radiographic progression rates prior to baseline (csDMARD period) and during TNFI treatment (TNFI period) were calculated [16]. Radiographic progression was defined as: a) a change in total Sharp score (TSS) >0; and b) a change in TSS greater than the smallest detectable change (SDC) [19]. Intra-observer intra-class correlation coefficient (one-way random effects model) (ICC) for baseline TSS was 0.96 and for TSS change was 0.35 [20]. SDC was 3.9 TSS units/year [17, 21].

### DXR-BMD

DXR is a computerized version of the traditional radiogrammetry, previously described in detail [22]. Software automatically detects regions of interest in the three middle metacarpal bones and estimates the bone mineral density (DXR-BMD) based on cortical thickness. Short-term coefficient of variation has been shown to be 0.28 % and smallest detectable difference 0.0046 g/cm [7, 23].

X-ray films from the reference cohort were analysed with the Pronosco X-posure™ v. 2.0 equipment (Sectra, Sweden). DXR-BMD of both hands were obtained and the mean used for analyses (available in 1485/2541 men/women as 48 men and 77 women were excluded due to missing or unsuitable x-rays). Patient x-rays in a digital format were analysed with the DXR-online system (Sectra, Sweden). The same algorithm was used for calculating DXR-BMD with the Pronosco X-posure™ and DXR-online systems [24].

**Fig. 1:**
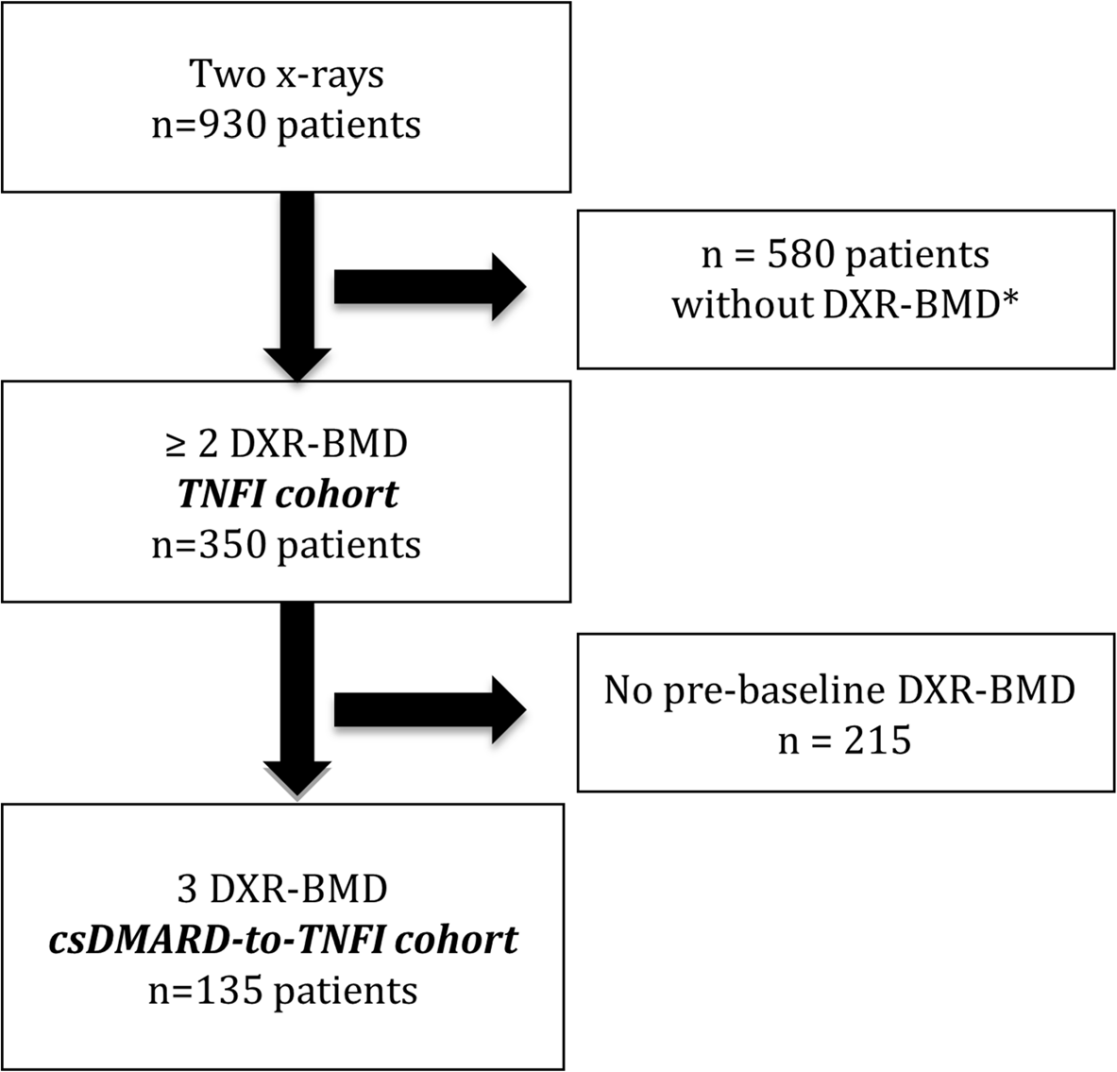
Patient disposition. *Patients were excluded if: a) DXR-BMD could not be analysed due to technical problems (insufficient exposure of x-rays, insufficient positioning of hands) or disease-related factors (prostheses, severe bone damage); or b) DXR-BMD change (i.e. hand bone loss) could not be calculated due to change in acquisition modality between original x-rays (e.g. x-ray film at baseline and digital image at follow-up) or large change in digital image post-processing between x-rays (edge enhancement and change in x-ray resolution). *csDMARD* Conventional synthetic disease-modifying anti-rheumatic drug, *DXR-BMD* Bone mineral density estimated by digital x-ray radiogrammetry, *TNFI* Tumour necrosis factor alpha inhibitors

As indicated in Fig. 1, DXR-BMD analysis or HBL calculation was not possible in a substantial number of patient x-rays due to methodological or disease-related issues. Both hands were measured if possible and the mean used for analyses. If a patient only had DXR-BMD of one hand at any one time point, all analyses were based on that hand (applicable in 38 patients). Three DXR-BMD measurements were available in 135 patients (csDMARD-to-TNFI cohort), while 350 patients had two DXR-BMD measurements (baseline and follow-up; TNFI cohort).

### Clinical data

Health assessment questionnaire (HAQ) score and disease activity score in 28 joints based on three variables (DAS28) including C-reactive protein (CRP) were obtained from DANBIO at three visits. For the 135 patients included in the csDMARD-to-TNFI cohort, the three visits were selected to be: closest in time to the pre-baseline x-ray (pre-baseline), closest to the date of TNFI initiation (baseline) and closest in time to the follow-up x-ray (follow-up)*.* For the 215 additional patients included in the TNFI cohort, the baseline and follow-up visits were selected in a similar manner, while the pre-baseline visit was the visit closest to 2 years prior to TNFI.

Patient files were reviewed and data on csDMARD and glucocorticoid treatment in the csDMARD and TNFI period registered.

To provide an estimate of inflammatory burden time-averaged CRP (available in 344 patients, median (interquartile range (IQR)) number of measurements 13 (7–20)), time-averaged DAS28, 28 swollen joint count (28SJC) and 28 tender joint count (28TJC) were calculated (available in 335 patients, based on 7 (5–11) measurements) [25].

### Statistical analyses

All analyses were performed with R (version 2.15.3) [26]. Analyses were two-sided with a significance level of 0.05.

#### Reference cohort

Linear regression models for the relation between age and DXR-BMD were fitted for men and women separately. Model fits were compared with the Akaike information criterion (AIC) for non-nested models and analysis of variance (ANOVA) for nested models. Standard graphical tests of model assumptions were performed (plots inspected for linearity, homoscedasticity and normally distributed residuals). From the final models estimated mean annual change in DXR-BMD were calculated for all years of ages from 18 to 89 in both sexes. These estimates constitute reference values for normal HBL/year in the present study.

#### Patients with RA

HBL is presented as annual absolute (g/cm^2^) and relative (%) change in DXR-BMD. Increased HBL in an individual patient was defined as a negative HBL/year exceeding the lower 95 % confidence interval (CI) of the normal HBL/year for the matching sex and year of age. For example, a female patient of 54 years would be said to have increased HBL if her HBL/year was lower than –0.0051 g/cm^2^ (Additional file 1: supplementary table). HBL was compared between csDMARD and TNFI periods by non-parametric analyses due to a skewed distribution of HBL.

Univariate logistic and linear regression were used to analyse the association between inflammatory activity (assessed with time-averaged CRP, DAS28, 28SJC and 28TJC) and increased and absolute HBL, respectively.

Correlation between HBL and radiographic progression was analysed with Spearman’s rho.

Possible predictors for increased HBL were analysed with univariate logistic regression, and significant variables (*p* < 0.10) included in a multiple logistic regression analysis with backward selection.

## Results

### Reference cohort

**Fig. 2:**
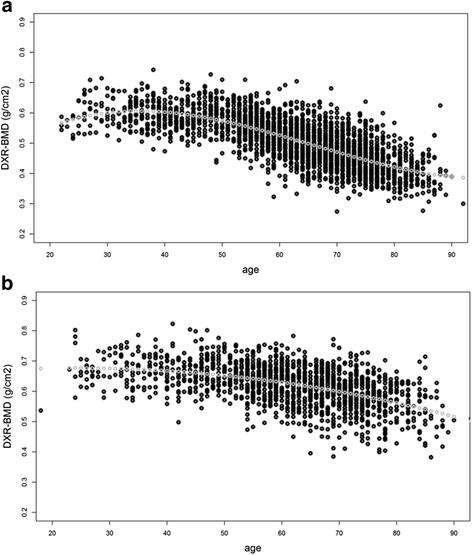
Distribution of DXR-BMD (g/cm^2^) in **a** 2541 Danish women and **b** 1485 Danish men. The *grey dotted* lines indicate regression lines fitted to the data from the reference cohort. *DXR-BMD* Bone mineral density estimated by digital x-ray radiogrammetry

Distribution of DXR-BMD in women and men in the reference cohort is presented in Fig. 2. In women, the best fitting model was DXR-BMD = 0.020 × age – 0.00040 × age^2^ + 0.0000021 × age^3^ (R^2^ = 0.54); in men, DXR-BMD = 0.0018 × age – 0.0000371 × age^2^ (R^2^ = 0.27). Estimated mean annual changes in DXR-BMD (i.e. normal HBL/year) in men and women derived from the models are presented in Additional file 1. Table 1 presents normal HBL/year averaged over 5-year age intervals (10-year intervals in the lowest and highest age groups). In men, the model estimated an increasing HBL/year from 35 years of age onwards reaching a maximum of –0.0047 g/cm^2^/–0.8 % per year at the age of 85. In women, an annual increase in DXR-BMD until 35 years of age was estimated, followed by a continuous HBL that accelerated between 55 and 70 years (greater than –0.0050/–1.0 %).

**Table 1:** DXR-BMD and estimated mean annual change in DXR-BMD (i.e. normal HBL/year) in 1485 Danish men and 2541 Danish women

			Absolute HBL/year		
Age (years)	n	BMD (g/cm^2^) (mean (SD))	Estimate	95 % Confidence interval	Percentage HBL/year
Men					
18–29	32	0.666 (0.06)	–0.00012	–0.00095 to 0.00071	–0.02
30–34	35	0.671 (0.06)	–0.00060	–0.00128 to 0.00007	–0.09
35–39	45	0.679 (0.05)	–0.00098	–0.00153 to –0.00042	–0.1
40–44	71	0.660 (0.05)	–0.00135	–0.00179 to –0.00090	–0.2
45–49	98	0.668 (0.05)	–0.00172	–0.00206 to –0.00138	–0.3
50–54	133	0.653 (0.05)	–0.00202	–0.00235 to –0.00184	–0.3
55–59	209	0.636 (0.05)	–0.00247	–0.00269 to –0.00225	–0.4
60–64	198	0.623 (0.06)	–0.00284	–0.00309 to –0.00259	–0.5
65–69	211	0.602 (0.06)	–0.00321	–0.00354 to –0.00288	–0.5
70–74	213	0.594 (0.06)	–0.00358	–0.00401 to –0.00316	–0.6
75–79	140	0.572 (0.06)	–0.00396	–0.00450 to –0.00342	–0.7
80–84	68	0.563 (0.06)	–0.00432	–0.00499 to –0.00367	–0.8
85–93	32	0.534 (0.08)	–0.00470	–0.00548 to –0.00392	–0.8
Total	1485	0.619 (0.07)			
Women					
18–29	41	0.597 (0.05)	0.00416	0.00268 to 0.00564	0.7
30–34	51	0.599 (0.04)	0.00123	0.00031 to 0.00213	0.2
35–39	84	0.601 (0.05)	– 0.00066	–0.00122 to –0.00009	–0.1
40–44	116	0.589 (0.04)	– 0.00224	–0.00260 to –0.00189	–0.4
45–49	131	0.592 (0.05)	–0.00351	–0.00379 to –0.00323	–0.6
50–54	185	0.569 (0.05)	–0.00437	–0.00470 to –0.00411	–0.8
55–59	322	0.541 (0.05)	–0.00513	–0.00543 to –0.00483	–0.9
60–64	311	0.513 (0.06)	–0.00548	–0.00575 to –0.00521	–1.0
65–69	430	0.486 (0.06)	–0.00552	–0.00577 to –0.00527	–1.1
70–74	394	0.460 (0.05)	–0.00525	–0.00559 to –0.00492	–1.1
75–79	307	0.440 (0.05)	–0.00468	–0.00525 to –0.00410	–1.0
80–84	123	0.411 (0.05)	–0.00380	–0.00472 to –0.00288	–0.9
85–93	46	0.400 (0.06)	–0.00261	–0.00397 to –0.00124	–0.7
Total	2541	0.505 (0.08)			

*BMD* Bone mineral density, *DXR-BMD* Bone mineral density estimated by digital x-ray radiogrammetry, *HBL* hand bone loss, *SD* Standard deviation

### Patients with RA

A patient disposition is shown in Fig. 1, while demographic, clinical, treatment and radiographic characteristics of included RA patients are summarised in Table 2. Patients with x-rays unsuitable for DXR-BMD had longer disease duration and more radiographic damage than patients included in the TNFI cohort, but had less functional disability. Other characteristics were similar between cohorts. In the csDMARD-to-TNFI cohort, the median (range) number of days from pre-baseline x-ray to baseline (TNFI initiation) was 607 (180–2989) days, from baseline to baseline x-ray 11 (90–866) days, and from baseline to follow-up x-ray 687 (198–1812) days.

**Table 2:** Demographic, clinical, treatment and radiographic characteristics of the patients included in the csDMARD-to-TNFI and TNFI cohorts

	csDMARD-to-TNFI cohort			TNFI cohort		Patients with radiographs unsuitable for DXR analysis	
Time point	Pre-baseline	Baseline	Follow-up	Baseline	Follow-up	Baseline	*p* value^a^
No. of patients	135	135	135	350	350	580	
Age (years)	55 (44–62)	57 (47–64)	59 (48–65)	56.5 (47–64)	58 (48–66)	57 (49–64)	0.44^*^
Women (%)	85	85	85	79	79	75	0.08^**^
Disease duration (years)	4.5 (1–10)	6 (4–12)	8 (5–14)	8 (4–14)	10 (6–15)	10 (5–18)	**0.004** ^*^
DAS28	4.3 (3.0–5.3)	5.3 (4.4–6.1)	3.1 (2.2–3.9)	5.3 (4.5–6.1)	3.0 (2.1–4.0)	5.4 (4.7–6.2)	0.24^*^
CRP (mg/l)	16 (8–33)	18 (8–40)	8 (4–14)	17 (8–35)	8 (3–14)	19 (8–42)	0.09^*^
IgM-RF positivity (%)	71	71	71	76	76	79	0.77^**^
Current smokers (%)	36	35	30	34	32	37	0.25^**^
HAQ score	NA	1.250 (0.750–1.750)	0.825 (0.250–1.375)	1.250 (0.750–1.750)	0.875 (0.250–1.375)	0.875 (0.250–1.500)	**<0.001** ^*^
Previous csDMARDs (n)	NA	3 (3–4)	–	3 (2–4)	–	3 (2–4)	0.26^*^
Length of TNFI period (days)	–	–	574 (405–759)	–	511 (381–695)	538 (397–761)	**0.03** ^*^
Concomitant MTX (%)	93	83	74	78	72	75	0.32^**^
Corticosteroid treatment (%)	–	88^c^	76^d^	71^c^	64^d^	57^d^	**0.03** ^**^
Prednisolone dosage (mg/day)^b^	–	1.32 (0.4–5.2)^c^	1.33 (0.4–3.8)^d^	1.8 (0.5–5.1)^c^	2.0 (0.5–4.6)^d^	2.7 (0.6–5.6)^d^	**0.02** ^*^
Type of TNFI (n (%))							
INF		101 (75)		218 (62)			
ETA		17 (12)		68 (20)			
ADA		18 (13)		64 (18)			
Treatment at follow-up (n (%))							
On first TNFI			79 (59)		216 (62)		
Switched from first TNFI			38 (27)		94 (27)		
Withdrawn from TNFI			18 (14)		40 (11)		
Sharp score (TSS units)	6 (0–21)	10 (1–29)	12 (2–31)	11 (2–34)	12 (3–35)	17 (3–53)	**0.001** ^*^
Sharp score (TSS units) (mean (SD))	16 (22)	19 (24)	20 (24.6)	22 (27)	23 (27)	36 (45)	**0.001** ^***^
Erosive disease (%)	70	76	77	81	81	83	0.55^**^
DXR-BMD (g/cm^2^) (mean (SD))	0.529 (0.09)	0.506 (0.10)	0.495 (0.10)	0.502 (0.10)	0.490 (0.10)	NA	

^a^Comparing TNFI cohort with the cohort of patients with radiographs unsuitable for DXR (values in bold are significant)

^b^All administered corticosteroids (peroral, intra-articular and intramuscular) were converted to a corresponding daily dosage of prednisolone in patients who had received corticosteroids at least once

^c^In the csDMARD period

^d^ In the TNFI period. Medians (interquartile range) are presented unless otherwise indicated.

^*^Mann-Whitney

^**^Chi-square

^***^Students *t*-test

*ADA* Adalimumab, *CRP* C-reactive protein, *csDMARD* Conventional synthetic disease-modifying anti-rheumatic drug, *DAS28* Disease activity score in 28 joints based on three variables including CRP, *DXR* Digital x-ray radiogrammetry, *DXR-BMD* Bone mineral density measured by digital x-ray radiogrammetry, *ETA* Etanercept, *HAQ* Health assessment questionnaire, *IgM-RF* Immunoglobulin M rheumatoid factor, *INF* Infliximab, *NA* not available, *SD* Standard deviation, *TSS* Total Sharp Score, *MTX* Methotrexate, *TNFI* Tumour necrosis factor inhibitor

#### HBL in the csDMARD-to-TNFI cohort

In the 135 patients in the csDMARD-to-TNF cohort, pre-baseline median (IQR) DXR-BMD was 0.545 (0.474–0.597) g/cm^2^ decreasing to 0.516 (0.441–0.578) g/cm^2^ at baseline (*p* < 0.001, Wilcoxon Signed Rank). At follow-up, DXR-BMD had further decreased to 0.504 (0.424–0.557) g/cm^2^ (*p* < 0.001, Wilcoxon Signed Rank). In the csDMARD period, 101 (75 %) patients had increased HBL while increased HBL was found in 79 (59 %) patients in the TNFI period (*p* = 0.004, McNemars test). Thirty-eight patients had an increased HBL in the csDMARD period that normalised in the TNFI period, while 16 patients had a normal HBL in the DMARD period but increased HBL in the TNFI period. Of the latter 16 patients, 6 patients continued their first TNFI treatment throughout follow-up, while three and seven patients, respectively, withdrew from TNFI treatment or switched to a different biological drug. Corresponding numbers for the 38 patients with normalized HBL during TNFI were 26, 5 and 7.

HBL was significantly lower in the TNFI period (–0.0051 g/cm^2^/year and –1.15 %/year) compared to the csDMARD period (–0.0082 g/cm^2^/year and –1.55 %/year; *p* < 0.001 for both comparisons, Wilcoxon Signed Rank).

**Fig. 3:**
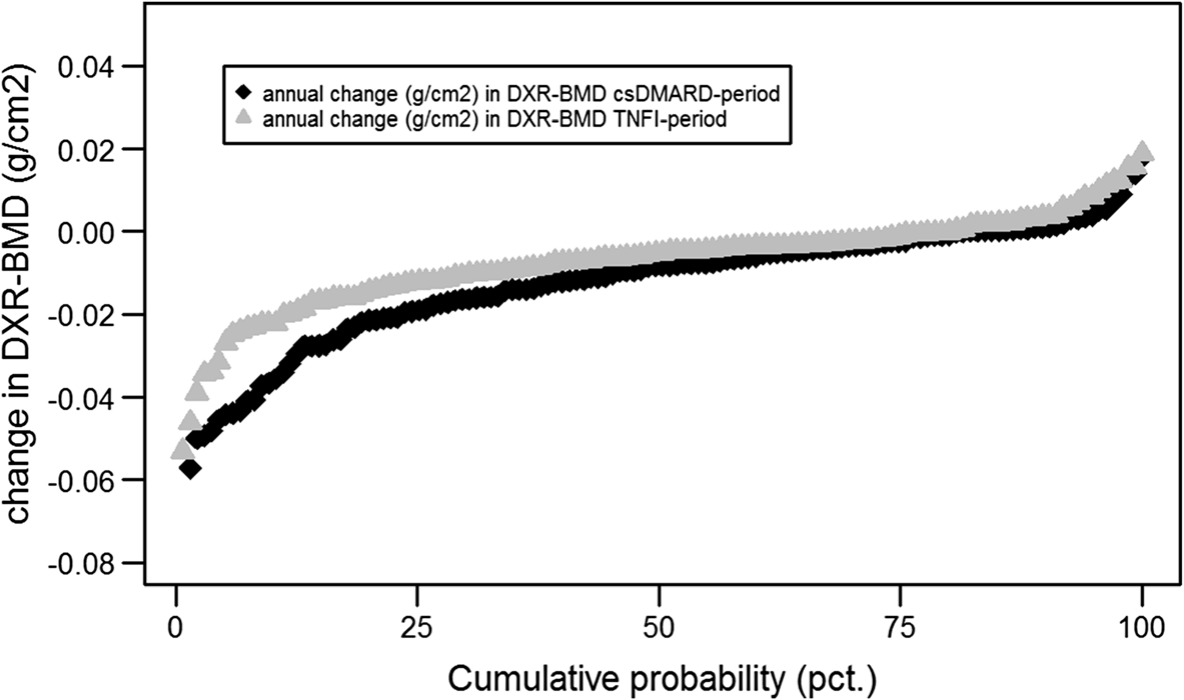
Cumulative probability plot illustrating HBL in each individual patient in the csDMARD period and TNFI period. The csDMARD period is the period between the pre-baseline and baseline x-rays, while the TNFI period is the period between baseline and follow-up x-rays (see Patients and methods for details). *csDMARD* Conventional synthetic disease-modifying anti-rheumatic drug, *DXR-BMD* Bone mineral density measured by digital x-ray radiogrammetry, *TNFI* Tumour necrosis factor inhibitors

Figure 3 illustrates a probability plot for HBL in the csDMARD and TNFI periods.

#### HBL and inflammatory activity during TNFI treatment

In the TNFI cohort (n = 350), increased HBL during TNFI was associated with time-averaged DAS28 (odds ratio (OR) 1.69 (95 % Confidence Interval 1.34-2.15/unit increase) *p* < 0.001) and all of its components: time-averaged 28SJC (OR 1.29 (1.15–1.46/joint increase), *p* < 0.001), time-averaged 28TJC (OR 1.11 (1.05–1.19/joint increase), *p* < 0.001) and time-averaged CRP (OR 1.02 (1.005–1.04/(mg/L) increase), *p* = 0.02). Similarly, absolute HBL was associated with time-averaged DAS28 (β-coefficient –0.0032 (–0.0020 to –0.0041) g/cm^2^, *p* <0.001) and all of its components: time-averaged 28SJC (–0.0008 to –0.002) g/cm^2^, *p* < 0.001), time-averaged 28TJC (–0.0004 (–0.00008 to 0.0007) g/cm^2^, *p* = 0.01) and time-averaged CRP (–0.0003 (–0.0002 to –0.00004) g/cm^2^, *p* < 0.001)) in linear regression analyses.

During TNFI treatment, 81 patients were in time-averaged remission (time-averaged DAS28 < 2.6) and 42 (52 %) of these patients had a normal HBL. In contrast, 98 (39 %) of the 254 patients not in time-averaged remission had a normal HBL (*p* = 0.04, Chi squared). Patients in time-averaged remission had a lower HBL than patients who were not (–0.0032 vs. –0.0058 g/cm^2^/year; *p* = 0.003, Mann-Whitney).

Time-averaged CRP was <10 mg/L during TNFI treatment in 174 patients and 84 (48 %) of these patients had a normal HBL, while 51 (36 %) of the 141 patients with a time-averaged CRP >10 mg/L had a normal HBL (*p* = 0.04, Chi squared).

#### HBL and radiographic progression

Median (IQR)/mean (standard deviation) rate of radiographic progression decreased from 0.6 (0.0–2.5)/1.9 (3.2) TSS units/year in the csDMARD period to 0.0 (0.0–0.8)/0.5 (1.7) TSS units/year in the TNFI period (*p* < 0.001, Wilcoxon Signed Rank, paired *t*-test). In the TNFI cohort the rate of radiographic progression was 0 (0–0)/0.5 (1.8) TSS units/year.

In the csDMARD-to-TNFI cohort (n = 135), 73 patients progressed radiographically (change in TSS >0) during csDMARD treatment and 56 (77 %) of these patients experienced increased HBL. Of the 62 patients who did not progress radiographically, 45 (72 %) had increased HBL (Chi squared = 0.72). In the TNFI period, 41 patients progressed radiographically and 28 of these patients (68 %) had increased HBL, while increased HBL was found in 51 of the 94 patients who did not progress radiographically (54 %; Chi squared = 0.18). Similarly, 64 % vs. 56 % of the patients who progressed/did not progress radiographically in the TNFI cohort (n = 350) had increased HBL (Chi squared = 0.18). Radiographic progression above the SDC (3.9) was found in 24 patients in the csDMARD-to-TNFI cohort in the csDMARD period, while six patients experienced progression in the TNFI period. As was the case for the cut-off of 0, no relation between increased HBL and radiographic progression above the SDC was found in either the csDMARD or the TNFI period (Chi squared = 0.7 and 0.4, respectively).

Correlations between TSS status scores and separate erosion score (ES) and joint space narrowing (JSN) scores with DXR-BMD were moderate, while correlations between TSS, JSN and ES changes with HBL were low (Table 3).

**Table 3:** Correlations between DXR-BMD and radiographic scores in the csDMARD-to-TNFI cohort

	DXR-BMD status				Hand bone loss	
n =135	Pre-baseline	Baseline	Follow-up		csDMARD period	TNFI period
JSN	–0.42 (<0.001)	–0.41 (<0.001)	–0.37 (<0.001)	JSN change	–0.09 (0.27)	–0.18 (0.002)
ES	–0.46 (<0.001)	–0.48 (<0.001)	–0.47 (<0.001)	ES change	–0.19 (0.02)	–0.13 (0.01)
TSS	–0.47 (<0.001)	–0.47 (<0.001)	–0.45 (<0.001)	TSS change	–0.21 (0.01)	–0.25 (0.003)

*p* values from Spearmans rho are shown in parentheses

*csDMARD* Conventional synthetic disease-modifying anti-rheumatic drug, *DXR-BMD* Bone mineral density estimated by digital x-ray radiogrammetry, *ES* Erosion score, *JSN* Joint space narrowing, *TNFI* Tumour necrosis factor alpha inhibitors, *TSS* Total Sharp score

#### Predictors of HBL during TNFI treatment

In univariate analyses of the TNFI cohort, high baseline DXR-BMD, longer disease duration, immunoglobulin M rheumatoid factor positivity and high DAS28 were associated with increased HBL, while age, HAQ score, CRP, sex, smoking status, type of TNFI, calendar year of treatment initiation and concomitant treatment with prednisolone and MTX were not. In the multivariable model, DXR-BMD (OR 1.005/mg increase (95 % CI 1.003–1.008)) and DAS28 (OR 1.43/unit increase (95% CI 1.15–1.81)) were independently associated with increased HBL. Fraction of explained variation (Nagelkerkes R^2^) was 0.12. In a model including time-averaged DAS28 during TNFI treatment, baseline DXR-BMD and DAS28 remained independent predictors of increased HBL (R^2^ increased to 0.20).

## Discussion

In this paper we have established reference values for hand bone mass measured by DXR-BMD. Based on this reference material we estimated sex- and age-related mean HBL/year and used this to define whether HBL was increased or not in individual RA patients. Our main finding was that the majority of patients initiating TNFI treatment in clinical practice had increased HBL and that normalisation of HBL during TNFI treatment was achieved only in a minority of patients.

The large reference cohort (n = 4026) allows precise estimation of sex- and age-related mean HBL/year. The reference cohort was randomly recruited and excluded individuals with inflammatory joint diseases. Individuals with other diseases affecting BMD (osteoporosis, kidney disease, etc.) were not excluded. Theoretically, this could result in overestimation of HBL in the reference cohort. However, a smaller Danish reference cohort excluded such individuals and no systematic differences between DXR-BMD in the two cohorts were found (data not shown) [27].

Based on the distribution of HBL in two early RA cohorts (EURIDISS and BARFOT), the manufacturer of DXR (Sectra, Sweden) defines moderately increased HBL as a negative change in DXR-BMD >0.25 mg/cm^2^/month and <2.5 mg/cm^2^/month (>0.003 g/cm^2^/year and <0.03 g/cm^2^/year) and highly increased HBL as negative change in DXR-BMD >2.5 mg/cm^2^/month (>0.03 g/cm^2^/year) irrespective of age and sex [6, 7, 10]. According to our reference values, normal HBL in women >50 years of age and men >70 years is >0.003 g/cm^2^/year (corresponding to moderately increased HBL according to the manufacturer), emphasizing the need for an improved definition of increased HBL. No data have demonstrated clinical relevance of the distinction between moderately and highly increased HBL, which is why we chose to simply dichotomise normal vs. increased HBL. The main limitation of our approach is the cross-sectional design of the reference cohort resulting in estimated mean values of HBL, not true HBL measured in individuals. This approach is an established method when no longitudinal studies are available, but should ideally be supplemented with longitudinal data [28]. Melton et al. compared rates of BMD loss by dual-energy x-ray absorptiometry (DEXA) estimated from cross-sectional baseline data with rates obtained from longitudinal assessment and found that cross-sectional data overestimated BMD loss in some skeletal sites (hip and spine) and underestimated the loss in others (radius and ulna) [29]. The background for these findings is unclear and the relevance for DXR is not known.

This study is the first to investigate HBL in established RA during csDMARD treatment and subsequent TNFI treatment. In the BEST and PREMIER trials, patients on MTX monotherapy had a relative HBL/year of –2.6 and –1.9 %, respectively, compared to –1.55 % in our cohort during csDMARD treatment [5, 13]. The higher HBL in the RCT monotherapy arms may be explained by frequent use of combination treatment and intra-articular injections in our cohort and the RCT inclusion criteria selecting patients with early and aggressive disease.

As expected, we observed a decrease in HBL (from –1.55 %/year to –1.15 %/year) after initiation of TNFI treatment. The latter is considerably higher than HBL in the combination treatment arms in BEST (–0.6 and –0.9 %/year), but lower than the –1.63 %/year in the combination arm of PREMIER, probably reflecting stricter inclusion criteria in PREMIER than in BEST and our observational study. An HBL of –0.45 %/year was found in 215 patients with established RA receiving different treatments in the Oslo RA register [3]. This considerably lower HBL may be explained by moderate baseline disease activity (DAS28 = 4.0) in contrast to high disease activity in our cohort (DAS28 = 5.3).

Baseline DAS28 was an independent predictor of increased HBL in our cohort in accordance with previous studies documenting a predictive value of baseline DAS28 for future HBL [3, 5, 13, 24]. Interestingly, baseline CRP was not predictive of increased HBL while time-averaged CRP during TNFI treatment was strongly associated with increased HBL. This suggests a key role of sustained inflammation in HBL supporting results from the BEST trial where differences in HBL between treatment groups disappeared after adjustment for changes in disease activity. A similar association is well established with regards to radiographic progression but has not previously been shown for HBL [17, 30].

Previously, older age (PREMIER) and postmenopausal status (BEST) has been shown to be predictive of HBL in linear regression analyses [5, 13]. In contrast, we found that older age was not associated with increased HBL. This difference stresses the importance of establishing reference data for normal HBL in different age groups and in both sexes.

In accordance with earlier studies, correlations between DXR-BMD and radiographic damage were moderate, and correlations between HBL and radiographic progression low [5–7, 22, 24]. Increased HBL was found in 110 patients with no radiographic progression during TNFI treatment, suggesting a higher sensitivity of HBL in detecting RA-related bone damage. However, we also identified 40 patients who progressed radiographically, but had normal HBL. This dissociation between the two types of bone damage was also seen in a study by Hoff et al. implying that the exact pathophysiological mechanisms may differ and that the traditional evaluation of x-rays cannot be substituted by DXR or vice versa [7, 31]. In our study, overall radiographic progression was low, reflected in a poor ICC for TSS change [32]. The SDC of 3.9 TSS units is comparable to SDCs reported in major RCTs of patients with established RA [33, 34].

A definite limitation of our study is that many patient x-rays could not be analysed. This is a general weakness of DXR, limiting generalisability of our findings as patients with the most advanced and aggressive disease were excluded. A potential limitation of our study is the timing of x-rays, as the selection criteria theoretically allow for a HBL during TNFI treatment to be calculated based on x-rays obtained 90 days prior to baseline and 90 days after baseline. This could lead to an underestimation of the effectiveness of TNFI treatment. However, only two patients in the csDMARD-to-TNFI cohort had a baseline x-ray that preceded TNFI start in combination with a follow-up x-ray less than 300 days after TNFI start, and this is why we consider this bias minimal.

A potential limitation was the lack of information on the use of anti-resorptive treatment in the cohorts. Since previous studies have found no influence of alendronate on HBL, and the more potent anti-resorptive treatments were only marketed in the last part of our study period, we consider this limitation to be of minor significance [9, 13].

## Conclusions

In this study, we established a reference material for DXR-BMD in the general population and from this reference material we estimated normal sex- and age-related HBL/year. Applying these reference values we found increased HBL in the majority of RA patients in a cohort from clinical practice initiating TNFI treatment. HBL was only normalised in a few patients during TNFI treatment, and more often in patients who were in time-averaged remission.

## Additional file

Additional file 1:
**Estimated mean annual change in DXR-BMD (i.e. normal HBL/year) in 2541 Danish women and 1485 Danish men.** A Table of the estimated mean annual changes in DXR-BMD (i.e. normal HBL/year) in men and women derived from the final models for each year of age from 18 to 89 years in the reference cohort. (DOCX 31 kb)

## Abbreviations

28SJC: 28 Swollen joint count
28TJC: 28 Tender joint count
AIC: Akaike information criterion
ANOVA: Analysis of variance
BMD: Bone mineral density
CI: Confidence interval
CRP: C-reactive protein
csDMARD: Conventional synthetic disease-modifying anti-rheumatic drug
DAS28: Disease activity score in 28 joints based on three variables
DXR: Digital x-ray radiogrammetry
DXR-BMD: Bone mineral density estimated by digital x-ray radiogrammetry
ES: Erosion score
HAQ: Health assessment questionnaire
HBL: Hand bone loss
ICC: Intra-observer intra-class correlation coefficient
IQR: Interquartile range
JSN: Joint space narrowing
MTX: Methotrexate
Nagelkerkes R^2^: Fraction of explained variation
OR: Odds ratio
RA: Rheumatoid arthritis
RCT: Randomised controlled trial
SDC: Smallest detectable change
TNFI: Tumour necrosis factor alpha inhibitors
TSS: Total Sharp score
