# Supplementary material for: Establishment of age- and sex-adjusted reference data for hand bone mass and investigation of hand bone loss in patients with rheumatoid arthritis treated in clinical practice: an observational study from the DANBIO registry and the Copenhagen Osteoarthritis Study
Source: Arthritis Res Ther. 2016 Feb 24;18:53. doi: 10.1186/s13075-016-0952-y (PMC4766711; doi:10.1186/s13075-016-0952-y)
Supplement: Additional file 1: — Estimated mean annual change in DXR-BMD (i.e. normal HBL/year) in 2541 Danish women and 1485 Danish men. A Table of the estimated mean annual changes in DXR-BMD (i.e. normal HBL/year) in men and women derived from the final models for each year of age from 18 to 89 years in the reference cohort. (DOCX 31 kb) [file 13075_2016_952_MOESM1_ESM.docx]

Additional file 1: Estimated mean annual change in DXR-BMD (ie. normal HBL/year) in 2,541 Danish women and 1,485 Danish men

| Women: estimated mean annual change in DXR-BMD (g/cm^2^) | | | | Men: estimated mean annual change in DXR-BMD (g/cm^2^) | | | |
| --- | --- | --- | --- | --- | --- | --- | --- |
| Age | estimated mean change | lower 95% CI | upper 95% CI | age | estimated mean change | lower 95% CI | upper 95% CI |
| 22 | 0.005910697 | 0.004072255 | 0.007749139 | 22 | 0.0001416 | -0.000776324 | 0.0010595230 |
| 23 | 0.00538565 | 0.00365712 | 0.00711418 | 23 | 0.00006059 | -0.000826341 | 0.000960564 |
| 24 | 0.00487291 | 0.003250897 | 0.006494923 | 24 | -0.00001362 | -0.0008764 | 0.000861646 |
| 25 | 0.004372477 | 0.002853568 | 0.005891385 | 25 | -0.00008783 | -0.000926505 | 0.000762774 |
| 26 | 0.00388435 | 0.00246511 | 0.00530359 | 26 | -0.000156354 | -0.000976659 | 0.000663952 |
| 27 | 0.00340853 | 0.002085496 | 0.004731564 | 27 | -0.000230842 | -0.001026868 | 0.000565184 |
| 28 | 0.002945017 | 0.001714694 | 0.00417534 | 28 | -0.00030533 | -0.001077136 | 0.000466476 |
| 29 | 0.00249381 | 0.001352665 | 0.003634956 | 29 | -0.000379819 | -0.00112747 | 0.000367833 |
| 30 | 0.00205491 | 0.000999363 | 0.003110458 | 30 | -0.000454307 | -0.001177875 | 0.000269261 |
| 31 | 0.001628317 | 0.000654733 | 0.002601902 | 31 | -0.000528795 | -0.001228359 | 0.000170769 |
| 32 | 0.001214031 | 0.000318706 | 0.002109356 | 32 | -0.000603283 | -0.001278931 | 0.0000689042 |
| 33 | 0.000812051 | -0.000088485 | 0.001632903 | 33 | -0.000677772 | -0.0013296 | -0.000029154 |
| 34 | 0.000422378 | -0.000327889 | 0.001172646 | 34 | -0.00075226 | -0.001380377 | -0.000124143 |
| 35 | 0.0000450 | -0.000638686 | 0.00072871 | 35 | -0.000826748 | -0.001431275 | -0.000222221 |
| 36 | -0.000320047 | -0.000941345 | 0.00030125 | 36 | -0.000901236 | -0.001482309 | -0.000320164 |
| 37 | -0.0006728 | -0.001236053 | -0.000109547 | 37 | -0.000975725 | -0.001533496 | -0.000417954 |
| 38 | -0.001013246 | -0.001523041 | -0.000503451 | 38 | -0.001050213 | -0.001584855 | -0.000515571 |
| 39 | -0.001341386 | -0.001802579 | -0.000880193 | 39 | -0.001124701 | -0.001636411 | -0.000612991 |
| 40 | -0.001657218 | -0.002074975 | -0.001239461 | 40 | -0.00119919 | -0.001688191 | -0.000710188 |
| 41 | -0.001960744 | -0.002340562 | -0.001580926 | 41 | -0.001273678 | -0.001740228 | -0.000807128 |
| 42 | -0.002251963 | -0.002599658 | -0.001904268 | 42 | -0.001348166 | -0.00179256 | -0.000903773 |
| 43 | -0.002530876 | -0.002852502 | -0.00220925 | 43 | -0.001422654 | -0.001845234 | -0.001000075 |
| 44 | -0.002797482 | -0.003099165 | -0.002495798 | 44 | -0.001497143 | -0.001898305 | -0.00109598 |
| 45 | -0.003051781 | -0.003339462 | -0.002764099 | 45 | -0.001571631 | -0.001951842 | -0.00119142 |
| 46 | -0.003293773 | -0.003572896 | -0.00301465 | 46 | -0.001646119 | -0.002005925 | -0.001286314 |
| 47 | -0.003523459 | -0.003798681 | -0.003248237 | 47 | -0.001720608 | -0.002060652 | -0.001380563 |
| 48 | -0.003740838 | -0.004015835 | -0.00346584 | 48 | -0.001795096 | -0.002116143 | -0.001474048 |
| 49 | -0.00394591 | -0.004223313 | -0.003668506 | 49 | -0.001869584 | -0.002172542 | -0.001566627 |
| 50 | -0.00394591 | -0.004223313 | -0.003668506 | 50 | -0.001944072 | -0.002230019 | -0.001658125 |
| 51 | -0.004138675 | -0.004420122 | -0.003857229 | 51 | -0.002018561 | -0.002288781 | -0.001748341 |
| 52 | -0.004319134 | -0.004778405 | -0.004196168 | 52 | -0.002093049 | -0.002349062 | -0.001837036 |
| 53 | -0.004643131 | -0.004938599 | -0.004347664 | 53 | -0.002167537 | -0.00241113 | -0.001923944 |
| 54 | -0.00478667 | -0.005085547 | -0.004487793 | 54 | -0.002242025 | -0.00247527 | -0.002008781 |
| 55 | -0.004917902 | -0.005218942 | -0.004616862 | 55 | -0.002316514 | -0.002541767 | -0.002091261 |
| 56 | -0.005036827 | -0.005338575 | -0.004735079 | 56 | -0.002391002 | -0.002610878 | -0.002171126 |
| 57 | -0.005143446 | -0.005444328 | -0.004842563 | 57 | -0.00246549 | -0.002682799 | -0.002248182 |
| 58 | -0.005237757 | -0.005536163 | -0.004939351 | 58 | -0.002539979 | -0.002757628 | -0.002322329 |
| 59 | -0.005319762 | -0.005614123 | -0.005025402 | 59 | -0.002614467 | -0.002835352 | -0.002393582 |
| 60 | -0.005389461 | -0.005678337 | -0.005100584 | 60 | -0.002688955 | -0.002915847 | -0.002462064 |
| 61 | -0.005446852 | -0.005729034 | -0.00516467 | 61 | -0.002763443 | -0.002998901 | -0.002527986 |
| 62 | -0.005491937 | -0.005766562 | -0.005217312 | 62 | -0.002837932 | -0.003084247 | -0.002591617 |
| 63 | -0.005524715 | -0.005791417 | -0.005258014 | 63 | -0.00291242 | -0.003171597 | -0.002653243 |
| 64 | -0.005545187 | -0.005804273 | -0.0052861 | 64 | -0.002986908 | -0.00326067 | -0.002713147 |
| 65 | -0.005553351 | -0.005806013 | -0.00530069 | 65 | -0.003061397 | -0.003351204 | -0.002771589 |
| 66 | -0.005549209 | -0.005797723 | -0.005300695 | 66 | -0.003135885 | -0.003442972 | -0.002828798 |
| 67 | -0.005532761 | -0.005780647 | -0.005284874 | 67 | -0.003210373 | -0.003535776 | -0.002884971 |
| 68 | -0.005504005 | -0.005756045 | -0.005251966 | 68 | -0.003284861 | -0.003629451 | -0.002940272 |
| 69 | -0.005462943 | -0.005724997 | -0.005200889 | 69 | -0.00335935 | -0.00372386 | -0.002994839 |
| 70 | -0.005409574 | -0.005688216 | -0.005130933 | 70 | -0.003433838 | -0.00381889 | -0.003048786 |
| 71 | -0.005343899 | -0.005645961 | -0.005041836 | 71 | -0.003508326 | -0.00391444 | -0.003102208 |
| 72 | -0.005265916 | -0.005598104 | -0.004933729 | 72 | -0.003582815 | -0.004010448 | -0.003155181 |
| 73 | -0.005175627 | -0.005544275 | -0.00480698 | 73 | -0.003657303 | -0.004106835 | -0.003207771 |
| 74 | -0.005073032 | -0.005484007 | -0.004662057 | 74 | -0.003731791 | -0.004203552 | -0.00326003 |
| 75 | -0.004958129 | -0.005416837 | -0.004499421 | 75 | -0.003806279 | -0.004300556 | -0.003312003 |
| 76 | -0.00483092 | -0.005342356 | -0.004319484 | 76 | -0.003880768 | -0.004397807 | -0.003363728 |
| 77 | -0.004691404 | -0.005260223 | -0.004122585 | 77 | -0.003955256 | -0.004495276 | -0.003415236 |
| 78 | -0.004539582 | -0.005170164 | -0.003908999 | 78 | -0.004029744 | -0.004592935 | -0.003466553 |
| 79 | -0.004375452 | -0.00507196 | -0.003678944 | 79 | -0.00410423 | -0.004690762 | -0.003517703 |
| 80 | -0.004199016 | -0.004965441 | -0.003432592 | 80 | -0.004178721 | -0.004788738 | -0.0035687 |
| 81 | -0.004010273 | -0.004850469 | -0.003170078 | 81 | -0.004253209 | -0.004886846 | -0.003619573 |
| 82 | -0.003809224 | -0.004726939 | -0.002891509 | 82 | -0.004327697 | -0.004985071 | -0.003670323 |
| 83 | -0.003595868 | -0.004594766 | -0.002596969 | 83 | -0.004402186 | -0.005083402 | -0.003720969 |
| 84 | -0.003370205 | -0.004453882 | -0.002286528 | 84 | -0.004476674 | -0.005181829 | -0.003771519 |
| 85 | -0.003132235 | -0.004304232 | -0.001960239 | 85 | -0.004551162 | -0.00528034 | -0.003821984 |
| 86 | -0.002881959 | -0.004145771 | -0.001618147 | 86 | -0.00462565 | -0.005378929 | -0.003872372 |
| 87 | -0.002619376 | -0.003978465 | -0.001260287 | 87 | -0.004700139 | -0.005477588 | -0.00392269 |
| 88 | -0.002344486 | -0.003802281 | -0.000886691 | 88 | -0.004774627 | -0.005576311 | -0.003972943 |
| 89 | -0.00205729 | -0.003617198 | -0.000497382 | 89 | -0.004849115 | -0.005675092 | -0.004023139 |
